# The Cognitive and Psychological Factors (Personality, Driving Behavior, and Mental Illnesses) As Predictors in Traffic Violations 

**Published:** 2017-04

**Authors:** Seyyed Salman Alavi, Mohammad Reza Mohammadi, Hamid Soori, Maryam Ghanizadeh

**Affiliations:** 1 Psychiatry and Psychology Research Center, Tehran University of Medical Sciences, Tehran, Iran.; 2 Safety Promotion and Injury Prevention Research Center, Shahid Beheshti University of Medical Sciences, Tehran, Iran.; 3 Kerman Universityof Medical Sciences, Kerman, Iran.

**Keywords:** *Traffic Violations*, *Humanistic Factors*, *Mental Disorders*, *Personality Characteristics*, *Driving Behavior*

## Abstract

**Objective:** Driving is a complex behavior and is affected by several factors. This cohort study aimed at representing the main determinants such as personality features, driving behavior, and mental illnesses on driving violation based on logistic regression.

**Method:** In this cohort study, 800 heavy truck and lorry drivers were recruited. Participants were selected from those individuals who referred to Imam Sajjad hospital in Tehran, Iran during fall 2013 to summer 2015. Manchester Driving Behavior Questionnaire (MDBQ), Big Five Personality Test (NEO Personality Inventory), and SADS (Semi-Structural Interview) were used in this study. After 2 years, we checked all traffic violations caused by human factors involving the drivers. SPSS Version 18 was used for data analysis; t test and logistic regression (OR) was also used.

**Results:** The findings of the present study revealed significant differences between the 2 groups of drivers (those who were and were not involved in driving offenses) with respect to controlling the effective and demographic variables. Moreover, it was found that depression and anxiety could increase the chance of accidents (2.4 and 2.7 accidents odds, respectively) (P = 0.04, P = 0.004). Furthermore, just neuroticism could increase 1.1 odds of traffic offences (P = 0.009), but other personality traits did not have an effect on the equation.

**Conclusion:** Consistent with previous examinations, some mental disorders may affect traffic violations. Considering the magnitude and sensitivity of driving behavior, it is essential to evaluate multiple psychological factors in drivers before and after receiving or renewing their driver’s licenses.

Driving is a complex behavior with various factors. Many authors have proposed theoretical models to help describe these factors (1).

Driving behavior patterns include a set of conscious and unconscious factors that are collectively called cognitive behavioral characteristics. These patterns play an important role in traffic violations or road crashes (2). Compared with the research that emphasized such factors as road engineering, technical status of the vehicle, etc. on driving violation or road accident, a very few researches have been conducted on human factors such as anxiety, depression, or any personality features on driving violations using cohort methodology (3).

In the traffic culture and international definitions, human factors refer to the factors such as driver’s mental status, personality traits, and mental disorders (mental illness or psychiatric disorder based on DSM-V). The main factors that cause traffic violations by humanistic factors are mental disorders, stupefacient factors, fatigue and drowsiness, disability, inappropriate system to obtain a driver's license, lack of necessary training to ensure safety issues, lack of surveillance of human resources, and driving style (3, 4).

Generally, the main factors in violent driving are behavioral and psychological factors that cause 95% of traffic violations (5).

Thus, the research hypothesis was that in addition to recognizing the important role of road factors and technical defects of the vehicles in the incidence of traffic violations and accidents, the human factors that have not yet been studied (personality features based on Big 5 Personality Traits, driving behavior, and mental illnesses based on diagnostic interview) should also be investigated. The current prospective study was conducted to confirm the following hypothesis: Cognitive- behavioral factors and mental illnesses can predict offence in truck and lorry drivers.

Moreover, we compared the driving behaviors and its subscales in 2 groups of drivers: those with violations and those without violations. Thus, designing appropriate models to identify factors affecting traffic violations seems to be unavoidable and necessary because in Iran traffic violations and risky behaviors while driving are a common problem, causing Iran to have the highest road accident rate among Asian countries. As described in the scientific literature, the drivers’ behaviors (such as lapses, errors, or violations) are the best predictor of accidents (7-10). In addition, in the present study, traffic violations or road crashes caused by human factors, and violations caused by road conditions or vehicle health is reported based on police database, were surveyed.

## Materials and Methods

***Study Site***

Addiction Diagnosis Center, Imam Sajjad hospital, Tehran, Iran

***Participants***

In this cohort Study, 800 heavy truck and lorry drivers who referred to hospital to take a drug test to receive or renew their driver’s licenses were recruited during fall of 2013 and they followed- up to summer 2015. Drivers who took part in this study were professional drivers. Because the drivers of heavy vehicles are at special risks compared with other drivers, they were selected for our study. 

***Study Design***

The main aim of this study was to identify the psychological factors that influence traffic slips (unfastened seatbelt, lack of respect for traffic signals, over speeding, improper overtaking, careless driving, distracted driving, illegal turns, weaving through traffic, and red light). In a cohort study, the association between variables can be analyzed better than other types of research methodologies (6).

In this study, convenience sampling method was used. At first every participant, filled in the questionnaire, then, the reviewers conducted a semi-structured interview with the participants. Next, each participant was followed for 2 years.

Contact information including phone number, address, and national identification number were taken from each participant. After the follow up, the drivers who were involved in violations or driving errors caused by human factors were identified and their driving violations were as follow: unfastened seatbelt, lack of respect for the traffic signals, over speeding, improper overtaking, reckless driving, distracted driving, illegal turns, weaving through traffic, running the red light, etc. To determine which e driver was involved in a violation, we asked them to inform us about their traffic violations in a 2- year period (2013-2015). During the research period, data were extracted from the police traffic databases and the databases with proved accuracy of information. Most data were attained by the video recording or traffic cameras. For each participant, violation records were sought for a 2- year period from the year when the first members of the cohort were eligible for inclusion criteria. All attendees were informed about the aims of the study and were required to provide consent for participation. In addition, we were convinced that all participants had regularly used large vehicles (trucks or buses). Ethical approval was acquired from Tehran University of Medical Sciences, Tehran, Iran. The interviewers distributed the questionnaires among the drivers and convinced them to complete all the questionnaires. Moreover, a clinical psychologist conducted the semi- structured interviews (SADS interview) with the participants to diagnose any mental disorder in them. The questionnaires included the Demographic Questionnaire, Manchester Driving Behavior Questionnaire (MDBQ), NEO Personality Inventory, and Substance Use and Smoking Inventory. Inclusion criterion was as follows: Male occupational drivers of truck and lorry vehicles (category C or D) aged 23 years or older, who referred to Imam Sajjad hospital. Exclusion criteria were as follow: Female drivers and drivers who suffered from severe mental disorders such as chronic psychosis, or dementia, and illiterate or uneducated participants who could not understand the questions. 

***Data Collection***

To collect data, we used the following standardized questionnaires: 

***The Demographic Questionnaire***

The demographic questionnaire included driver’s age, level of education (based on dichotomous variable), driving years, overall mileage per month, marital status, time taken to obtain the driver's license, and the amount of occurred accidents caused by human factors. All attendees had a middle- or low- socioeconomic status. 

***Manchester Driving Behavior Questionnaire (MDBQ)***

This scale was designed and compiled by Rissen et al. in the Psychology Department of Manchester University (11).

It emphasizes that errors and violations have different psychological reasons and correction methods and so should be discriminated by the researchers. Nowadays, MDBQ is being used as a popular instrument to assess driving behaviors. This questionnaire contains 50 questions with Likert scale, ranging from 0 to 5. Questions have 2 different aspects. One aspect is the type of behavior, and the other relates to the number of risk posed to other drivers. Abnormal behaviors consist of lapse errors, slips, deliberate violation, and unintentional violation. These behaviors are classified as follow: 

1) Behaviors that pose no hazard to others and just give a feeling of comfort (low risk probability)

2) Behaviors with moderate risk probability

3) Behaviors that certainly put others at risk (high-risk probability).MDBQ have acceptable psychometric properties. Parker and Reason (1995) have obtained a correlation coefficient of 0.81 for errors and 0. 75 for violation in another reliability research conducted on 80 helmsmen with a 7-week interval (12).

Iliescu and Sârbescu (2013) reported that the factor reliability of DBQ is from .62 to .78, which is satisfactory; and they regarded the DBQ as a valid and reliable tool for assessing driving behavior (13).

Moreover, Oreizi (2015) reported that the Iranian version of DBQ has acceptable reliability and validity. In their study, factor reliability was from 0.65 to 0.81(14). In addition, Alavi et al. (2016) reported that the Iranian version of MDBQ has adequate reliability and validity. In their study, they extracted 6 factors, and the internal reliability of factors was from 0.65 to 0.75. The external reliability of MDBQ (test-retest correlations) was 0.56 and of split- half was 0.77(15).

***Personality Test (NEO Personality Inventory)***

To measure personality, we used the Persian prescription of the NEO- Five Factor Inventory. This scale contains 60 items and is scored from 0 to 4 (0 = fully disagree to 4 = fully agree). Each question inquires about the main 5 personality dimensions including neuroticism (N), extraversion (E), openness to experience (O), agreeableness (A), and conscientiousness (C), respectively. These factors covered 12 questions, with each scale scoring from zero to 48 (16).

The validity and reliability of the Persian version of NEO personality inventory have been approved in various studies. For instance, Yadollahi et al. (2014) have noted that the NEO has acceptable reliability (Cronbach’s α between 0.69-0.83)(17).

***Smoking and Substance Abuse Questionnaire***

This questionnaire consists of 12 items, with a Likert spectrum (5 point), and it surveys the dosage of the drugs, stimulants, hallucinogens, and alcohol during the past year. 

Rahimi- Movaghar et al. (2014) have cited that this questionnaire has acceptable reliability and validity. In their study, its inter-rater reliability was evaluated in the general population and showed a good reliability for smoking and alcohol use (18).

Due to the lack of financial and human facilities, we surveyed substance abuse through Smoking and Substance Abuse Questionnaire, the individuals whom we suspected of addiction, and police addiction checks via urine test or breath test.

***Psychiatric Interview (SADS)***

Clinical psychologists conducted the semi-structured interview to diagnose any mental disorder in the drivers. This interview assessed psychiatric disorders as follow: psychiatric problems; mood disorders including depression, mania and hypomania, substance abuse, and dependence; psychotic symptoms such as hallucination and delusions; anxiety disorders; somatization disorders; suicide thoughts; posttraumatic stress disorders (PTSD); dissociative disorders; epilepsy disorder; Alzheimer’s disease; and mental retardation.

The validity and reliability of SADS have been cited in several researches. Simpson SG et al.(2002) reported that the Cohen Kappa Coefficient of mania, hypomania, and depression was calculated to be 0.83, 0.72 and 1, respectively (19). Mohammadi et al. (2005) have reported that the Persian version of SADS has adequate validity and reliability (20). Clinical psychologists who were expert in diagnosing and treating mental disorders conducted the diagnostic interviews. 

Prior to completing the questionnaires, the participants were provided with a brief description of the study goals and an explanation on how to complete the questionnaires. In addition, to inspire attendees to report their traffic violations accurately, they were assured that data gathering was anonymous and their responses would be kept confidential. The questionnaires took almost 20 minutes to complete.

Data were analyzed using SPSS software Version 18.0. Also, descriptive statistics were used to demonstrate demographic data. To compare driving behavior and its subscales, we used Independent t test analysis. Logistic regression (computing the odds ratio and 95% CI) was used to assess the association of the main variables including age, education, some personality disorders, and personality characteristic of drivers on the likelihood of traffic violations. The effective factors on traffic violations were determined using logistic regression analysis. P-value less than 0.05 was considered as statistically significant.

All variables including Big 5 Personality Features, mental illnesses, and those demographic characteristics collected from the questionnaires, which were likely to have an impact on traffic violations, were identified for data analysis. Logistic analysis was conducted for each variable in the univariable analysis, and crude OR was also estimated; in the next step, the variables with p-value of <0.2 were selected and imported into the logistic regression analysis. Then, those variables that had a meaningful role in the model with a p-value of <0.05 were interpreted with adjusted OR. Moreover, Hosmer-Lemeshow x2 test was used to support the mensuration of the models (P value>0.01, x2<20).

## Results

In this cohort study, 800 drivers took part, whose ages ranged from 24 to 81 years, With an average of 46.7± 11.17 years (mean ± SD).

**Table1:** Summary of the Demographic Information from 800 Truck and Bus Participants

**Variable**		**Number of ** **Participants**	**Percentage (%)**
**Age(years)**	≤30	87	10.9
	31-40	160	20
	41-50	232	29
	51-60	247	30.9
	61-70	65	8.1
	71-80	8	1
**Marital status**	married	654	81.7
	single	146	18.3
**Education **	Diploma or less	730	91.2
	College education	70	8.8
**Driving history** *	1-10 years	185	23.4
	11-20	224	28.3
	21-30	230	29
	31 years or older	153	19.3
**Number of accidents (history of accident)**	1	132	16.6
	2	72	9
	3	27	3.4
	4	13	1.6
	5	8	1
	6	1	0.1
	7	1	0.1
	8	1	0.1
**At fault accident that samples had during the ** **past 5 years **	1	122	15.2
	2	39	4.9
	3	6	0.8
	4	1	0.1
	5	2	0.2

* Driving history: amount of years of driving per driver

**Table2:** The Mean, Standard Deviation (SD), and Comparison of Driving Behavior and Its Subscales in the Two Groups of Drivers; with Violation and Without Violation

						**%95 Confidence Interval ** **of the Difference**	
**Driving Behavior and Its** **Subscales**	**Violation History**	**Mean(±SD)**	**t**	**df**	**P-value**	Lower	Upper
**Driving behavior** *	no	16.4(13.12)	2	776	0.05	-5.26	-1.35
	yes	19(11.44)					
**Slips**	no	7.63(6.56)	-2.5	781	0.015	-3.42	-1.50
	yes	8.53(5.21)					
**Deliberate violation**	no	5.1(5.2)	-1.5	788	0.12	-2.1	0.25
	yes	6.1(5.1)					
**Laps error**	no	2.3(2.4)	-2.1	789	0.04	-1.3	-0.01
	yes	2.8(2.1)					
**Unintentional violation**	no	1.3(1.6)	-1.3	794	0.17	-0.61	0.11
	yes	1.6(1.6)					

* Driving Behavior: Scores of Driver's Driving Behavior Based on MDBQ

**Table 3:** Odds Ratios (95% CI) for Traffic Infringements in in truck and bus drivers (n = 800)

**Variables**	**Univariate Analysis**			**Multivariate Analysis**		
	**OR(crude)**	**CI (95%)**	**P-value**	**OR(adjusted)**	**CI (95%)**	**P-value**
Age	0.98	0.96-1.1	0.14	0.99^*^	0.96-0.99	0.05
Education	1.2	0.86-1.2	0.2	1.1	0.82-1.4	0.50
History of accident	1.2	1.1-1.4	0.02	1.2	0.98-1.4	0.07
Depression	2.9	2.1-7.2	0.018	3.1*	1.3-8.1	0.01
Obsession	3.9	1.2-13.1	0.02	4.2*	1.2-15.9	0.03
Antisocial personality	8.9	1.7-44.8	0.008	6.4*	1.2-35.9	0.03
Driving behavior	1.1	0.99-1.1	0.09	0.99	0.98-1.03	0.85
Agreeableness	1.08	1.03-1.14	0.002	1.1*	1.03-1.15	0.003
Openness to experience	0.96	0.92-1.05	0.07	0.96	0.91-1.08	0.21
Consciousness	0.98	0.94-1.02	0.19	1.5	0.95-1.04	0.98

OR adjusted: Odds Ratio

CI: Confidence Interval

All the respondents were male, 654(81.7%) were married, 730 (91.2%) held a secondary school degree or less, and 70 (8.8%) were educated. Table 1 summarizes some characteristics of the participants based on the demographic questionnaires. Table 2 demonstrates the mean, standard deviation (SD), and comparison of the driving behavior and its subscales between the 2 groups of drivers, with and without driving violation.

The results of the independent t test examination showed that the means of the 2 groups differed significantly in driving behavior, slips, and laps error (p-value < 0.05), but no significant differences were found between the 2 groups in deliberate violation and unintentional violation (p-value>0.05). Table 3 demonstrates the results of the logistic regression for traffic infringements. The main variables that influenced the traffic violations were age, depression, obsession, antisocial personality, and agreeableness.

Age significantly decreased the chance of traffic violation (OR = 0.99, 95% CI, 0.96-0.99).

Based on the results of the logistic regression, depression increased the odds of violation (OR = 3.1, 95% CI, 1.3-8.1).

In addition, among of anxiety disorders, obsession increased the chance of traffic violation (OR = 4.2, 95% CI, 1.2-15.9).

Education, history of accident, and driving behavior were not significant in the equation; and among the personality traits, agreeableness was 1.1 times more likely to increase the odds of traffic violations (95% CI, 1.03-1.15), and other personality factors did not seem to have an effect on the odds of traffic violations. Moreover, the results of the multivariable analysis revealed that antisocial personality increased the chance of traffic violation by 6.4 times.

Furthermore, because none of the participants mentioned any mental disorders, such as smells or visual delusion, psychosis symptoms, epilepsy, panic disorder, phobia, fugue, or other mental problems, we did not enter any of them into the equation.

## Discussion

The present study aimed to survey the human factors correlate with traffic contraventions.

Our study revealed that age was associated with decreasing in odds of traffic infringements, meaning that young drivers exhibit more risky driving behaviors than the older.

This result is consistent with most research performed in this field like the studies that have been quoted by Fernandes et al.(21), Clinton et al. (22),Tronsmoen (23), and Vaez and Laflamme (24). In these studies, they argued that when drivers are young, they drive too fast and perform high-risk behaviors due to internal excitement and lack of proper skills. These studies also revealed an inverse correlation between the amount of risky driving and driving experience like a research conducted by Lin in Taiwan reported that overtaking is decreased by increase in experience (25, 26).

The results revealed that education did not increase or reduce the chance of traffic violations. Despite these results, some studies found a relationship between education and driving safely; for instance, Habibi, Haghi and Maracy (2014) reported that educational level is an effective factor upon drivers understanding, while driving in traffic flows. Drivers with adequate education have more logical perception of environmental conditions and dangerous agents, and they pay more attention to traffic boards and blocks (27). However, risk taking behaviors among those with college education is significantly more than those who are under diploma. Similar results were reported by Haghshenas et al. In their study, they cited that safe driving is not associated with educational level (28). Thus, it might be concluded that the inconsistency of our results with those of the previous studies may be due to the proper sample size or educational level of the attendees. In our study, most of our sample had a diploma or less, and this impacted the results.

In the present study, no significant association was found between traffic violation and history of accidents. Despite these findings, some studies showed that upon the impact of an accident history on risk taking in young drivers, they found that those with accident history had a higher risk driving behavior (29).The inconsistency of our results with those of the previous researches may be due to the utilizing different sample groups and using self-reported questionnaire. Sometimes in our country, reporting accident history is considered as a taboo and this issue can affect the results.

The results also revealed that there were differences between means of the two groups in driving behavior, slips, and laps error. Recent researches have shown that behavioral factors including driving behavior and acceptance of higher levels of risk contribute to an enhanced risk of road crashes (30, 31). These studies highlight the need to improve drivers’ safe behaviors as the major aim for traffic safety interventions (32). In addition, safer behavior in driving is the result of right attitude and improved knowledge on driving (33). Mirzaeiet al. argued that safer behavior in driving is not attained unless in light of a safer attitude of drivers (34). Therefore, understanding of a greater behavioral and cognitive control may help drivers who represent the category at the highest odds of traffic violations and accidents. As stated by World Health Organization (WHO), the cause of road collisions has been mainly cognitive- behavioral and could be largely prevented by modifying personal and social behaviors (35).

Based on the results of the logistic regression, depression increased the chance of violation. Previous studies on risky driving indicated that neuropsychological illnesses including depression or ADHD (attention deficit hyperactivity disorder) also increase the chance of impaired driving (36). Another research found a positive and robust correlation between depression and risky driving behavior, especially in injury accidents and driving while plastered (37).Willemsen et al. (2008) reported that negative emotions represent a confusion, which can contribute to the cognitive processes necessary for driving safety, and thus enhances the chance of violations. It seems that drivers with depression have difficulty controlling negative emotions such as impulsive behavior or suicidal thoughts, which are reflected in speeding, less safety when changing lanes on the highway, and a greater probability of an accident following an unexpected event. Depression was also correlated with psychiatric drugs consumptions and side effects of antidepressants including drowsiness, decentralization, dizziness, weakness in vision, and fatigue (38) that contributed to traffic violations.

The results demonstrated that anxiety disorders increased 4.1 times the probability of traffic violations in bus and truck drivers. Previous reports demonstrated a significant association between anxiety and depression with offences. For example, Asghari et al. (2015) reported that there was a negative correlation between anxiety and driving psychology; thus, it may be concluded that anxiety and aggression negatively affect traffic psychology (39). Moreover, compulsive behaviors are usually associated with skepticism and dullness, and this leads to a person’s inability to take appropriate decisions in driving and may increase traffic statistics.

Overall, we can conclude that mental health is the predictor that has been surveyed in safety driving. It is believed that the drivers who suffer from mental disorders may jeopardize the health of themselves and others with their driving.

Our study revealed that among the personality traits, agreeableness increased the chance of violations, but other personality factors did not seem to have an effect on the chance of traffic violations. These results are both compatible and incompatible with those of previous studies. Wang, Rau, and Solvency’ study (2010) emphasized the factor of vision and personality traits in road accidents (40). Another research also revealed a positive association between the neuroticism index with a variety of errors and illegal acts in driving behavior, which confirms the results of this study (41).

Furthermore, Guo et al. (2016) reported that younger drivers scored higher on extroversion and agreeableness and lower on consciousness and accident involvement (42).

Also, they reported that higher agreeableness and lower consciousness were significantly correlated with higher risky driving behavior (42).

In contrast, Dahlen and white (2006) noted that conscientiousness is also negatively associated with risky driving (43).

Our results may be incompatible with those of the mentioned study due to several reasons: different samples in the studies, personality type, and the effects of other variables including type of license and history of driving. In the noted study, the participants were young drivers, but in our study, the drivers were selected from all age ranges, and this might have affected our results. Although friendly individuals respect traffic laws and regulations, violation of the law might be done by anyone with any personality trait in the future, thus, research should emphasize the role of personality characters in traffic violations.

Our results revealed that antisocial personality more likely enhance the odds of violations. These results are consistent with those of previous studies. For instance, Brown et al. (2016) cited that antisocial behavior is associated with risky driving (RD); and Vassallo et al. (2016) have reported that the most compatible correlates of hazardous driving patterns were antisocial personality or antisocial behavior (44, 45). It can be concluded that antisocial behavior is associated with risky behaviors such as alcohol drinking, high speed, and disregarding traffic rules, and interaction between these factors increases the likelihood of violations and traffic accidents. 

The complexity of individuals’ behavior comes from various factors including cultural, educational, economic, social factors, and mental health (46), which can affect the driving or traffic behavior of bus and truck drivers and may play an essential role in their behavior. On this topic, Rad et al. (2016) have reported that reckless driving such as speeding and violation of traffic laws are major risk factors for crashes in the South East of Iran. This highlights the need for educating the drivers along with enforcing traffic laws to reduce motor vehicle accidents in the future (47).

## Limitations

This study had several inherent limitations. First, the data were collected over a very short time (about 2 years), and administering the questionnaires (DBQ and NEO) had restrictions. Furthermore, the procedure for selecting the sample did not allow us to generalize the results to other populations such as category A & B (motor cycle and automobile) drivers or female drivers. Finally, illiterate or uneducated participants were excluded from our study and this could have influenced the results.

## Conclusion

The outcomes of this cohort study provided evidence that some mental disorders or personality traits may contribute to an increased chance of traffic offences. We also found that under-reporting of some mental disorders or some personality traits may be more frequent in some types of traffic violations or crashes than others.

Unsafe driving has recently been recognized as an illegal act that threatens road traffic safety. Nevertheless, this was the first prospective study to be conducted in Iran to survey the causes and investigate the cognitive-behavioral factors of traffic infraction. The data in this study could serve as a basis for future research projects in our country. These results may help design plans to improve driving safety in Iran. Moreover, using the data of the present study, we can manage the percentage of traffic violation in drivers. The novelty and strengths of this project were its prospective nature (cohort design) and a diagnostic semi-structured interview of all the samples to diagnose mental illnesses.

The combination of neuropsychobiological factors including impulsivity, negative emotions (such as suicidal ideation), and sensation seeking, predicted driving violations. Moreover, the finding of this study was compatible with the view that personality characteristics such as agreeableness can modulate the behavioral and emotional state of the driver, which in turn, is associated with higher rates of violations.
